# The burden of sepsis-associated mortality in the United States from 1999 to 2005: an analysis of multiple-cause-of-death data

**DOI:** 10.1186/cc7733

**Published:** 2009-02-27

**Authors:** Alexander Melamed, Frank J Sorvillo

**Affiliations:** 1 Keck School of Medicine of the University of Southern California, 1975 Zonal Avenue, Keith Administrative Building, Room 100-B, Los Angeles, CA 90089, USA; 2 Department of Epidemiology, School of Public Health, University of California, Los Angeles, CA 90095, USA

## Abstract

**Introduction:**

Sepsis is the 10th leading cause of death in the United States. The National Center for Health Statistics' multiple-cause-of-death (MCOD) dataset is a large, publicly available, population-based source of information on disease burden in the United States. We have analysed MCOD data from 1999 to 2005 to investigate trends, assess disparities and provide population-based estimates of sepsis-associated mortality during this period.

**Methods:**

Sepsis-associated deaths occurring in the United States from 1999 to 2005 were identified in MCOD data using *International Classification of Disease, 10th Revision *(ICD-10) codes. Population-based mortality rates were calculated using bridged-race population estimates from the National Center for Health Statistics. Comparisons across age, sex and racial/ethnic groups were achieved by calculating mortality rate ratios.

**Results:**

From 1999 to 2005 there were 16,948,482 deaths in the United States. Of these, 1,017,616 were associated with sepsis (6.0% of all deaths). The age-adjusted rate of sepsis-associated mortality was 50.37 deaths per 100,000 (95% confidence interval (CI) = 50.28 to 50.47). There were significant disparities in sepsis-associated mortality in race/ethnicity and sex groups (*P* < 0.0001). After controlling for age, Asians were less likely than whites to experience sepsis-related death (rate ratio (RR) = 0.78, 95% CI = 0.77 to 0.78), while Blacks (RR = 2.24, 95% CI = 2.23 to 2.24), American Indians/Alaska Natives (RR = 1.24, 95% CI = 1.24 to 1.25) and Hispanics (RR = 1.14, 95% CI = 1.13 to 1.14) were more likely than whites to experience sepsis-related death. Men were at increased risk for sepsis-associated death in all race/ethnicity categories (RR = 1.27, 95% CI = 1.27 to 1.28), but the degree of increased susceptibility associated with being male differed among racial/ethnic groups (*P* < 0.0001). Although crude sepsis-associated mortality increased by 0.67% per year during the study period (*P* < 0.0001), the age-adjusted mortality rate decreased by 0.18% per year (*P* < 0.01).

**Conclusions:**

The rapid rise in sepsis mortality seen in previous decades has slowed, but population ageing continues to drive the growth of sepsis-associated mortality in the United States. Disparities in sepsis-associated mortality mirror those previously reported for sepsis incidence. Sepsis in Asians, Hispanics and American Indian/Alaska Natives should be studied separately because aggregate measures may obscure important differences among these groups.

## Introduction

Sepsis is the 10th-leading cause of death in the United States, and one of only two infectious conditions listed in the leading 15 causes of death [1]. Sepsis incidence and mortality have increased over the course of several decades [2-4]. In addition to being common and often lethal, sepsis is costly, with an annual economic burden estimated at $16.7 billion [5].

Since 1992, sepsis has been defined by consensus as a systemic inflammatory response syndrome of infectious origin [6]. The failure of one or more organ systems or the occurrence of hypoperfusion in conjunction with sepsis is considered to be severe sepsis. Severe sepsis accompanied by hypotension is septic shock [7]. Death in septic patients has not been explained by autopsy studies, but it has been suggested that the cause of death is usually multiple organ failure [8,9].

Known risk factors for developing sepsis include advanced age, male gender and non-white race [5,10]. Comorbidities commonly associated with the condition include HIV infection, cancer, cirrhosis, alcohol dependence and pressure ulcers [11-16].

A number of recent studies have used administrative datasets to assess the burden and epidemiological features of sepsis [2,4,5,17-20]. Angus and colleagues used discharge records from a multi-state sample of non-federal hospitals to assess the incidence, outcome and economic burden of severe sepsis in the United States for the calendar year of 1995 [5]. Dombrovskiy and colleagues have employed discharge data from the New Jersey State Inpatient Database and the Nationwide Inpatient Sample to investigate trends and disparities in sepsis as well as severe sepsis on the state and national level [2,18,19]. Martin and colleagues [4] used the National Hospital Discharge Survey to quantify sepsis over a 21-year period, while Esper and colleagues [17] used the same data to probe at the role of comorbidity in sepsis disparities.

Few population-based sources of data can be used to investigate the burden of sepsis-associated mortality on a national level. To date, investigators have relied on samples of hospital discharge data for such estimates [2,4,5,17-20]. These data are weighted to extrapolate to national-level estimates and are therefore particularly vulnerable to sampling bias. Furthermore, mortality estimates from hospital data cannot be population based because sepsis-associated deaths may occur in non-hospital settings.

This study assessed sepsis-associated mortality using United States multiple-cause of-death (MCOD) data. Although MCOD data has been used to estimate national-level mortality rates for a variety of health conditions [16,21-25], we are not aware of previous studies that used this source of data to address sepsis. We have examined MCOD data from 1999 to 2005 to determine population-based estimates, trends and disparities in sepsis-related mortality.

## Materials and methods

We obtained MCOD data for sepsis-associated deaths occurring from 1999 to 2005. The study period was selected because 2005 was the most recent year for which data were available, and because MCOD coding practices changed between 1998 and 1999, with 1999 representing the first year that the data were coded according to the *International Classification of Disease, 10^th ^Revision *(ICD-10) [26]. MCOD data are abstracted from death certificates by the National Center for Health Statistics [27]. This study relied on publicly and de-identified data on deceased individuals, and consequently does not constitute research with human subjects according to Title 45, part 45, of the Code of Federal Regulations [28]. The University of Southern California exempts such research from Institutional Review Board oversight [29].

The underlying cause of death is "the disease or injury which initiated the train of morbid events leading directly or indirectly to death or the circumstances of the accident or violence which produced the fatal injury" [30]. National Center for Health Statistics employs underlying cause of death to report national mortality statistics. Sepsis is known to affect the elderly and other populations with high rates of chronic conditions which predispose them to infection [5,10,12-16]. For patients with underlying pathologies, sepsis may be a necessary condition in the causal pathway leading to death, but may not be listed as the underlying cause of death. Similarly, in cases where sepsis results from nosocomial infection, the original reason for hospitalisation, rather than sepsis, is often listed as the underlying cause of death. Consequently, analyses restricted only to decedents with sepsis listed as the underlying cause of death will significantly underestimate the true burden of sepsis-associated mortality.

Sepsis-related death was defined as a death where any of the following ICD-10 codes appears in any field of the death certificate: A40.0 (septicaemia due to streptococcus, group A), A40.1 (septicaemia due to streptococcus, group B), A40.2 (septicaemia due to streptococcus, group C), A40.3 (septicaemia due to streptococcal pneumonia), A40.8 (other streptococcal septicaemia), A40.9 (streptococcal septicaemia, unspecified), A41.0 (septicemia due to *Staphylococcus aureus*), A41.1 (septicaemia due to other specified staphylococcus), A41.2 (septicaemia due to other unspecified staphylococcus), A41.3 (septicaemia due to *Haemophilus influenzae*), A41.4 (septicaemia due to anaerobes), A41.5 (septicaemia due to other Gram-negative organisms), A41.8 (other specified septicaemia), A41.9 (septicaemia, unspecified), A02.1 (salmonella septicaemia), A22.7 (anthrax septicaemia), A26.8 (erysipelothrix septicaemia), A32.7 (listerial septicaemia), A42.7 (actinomycotic septicaemia), B00.7 (herpesviral septicaemia), and B37.7 (candidal septicaemia). Like other researchers who have investigated sepsis utilising administrative datasets, we used ICD codes for septicaemia to identify sepsis-associated deaths [2,4,17-19].

For sepsis-associated deaths we analysed age, sex, race, ethnicity, year-of-death, place-of-death and any other medical conditions mentioned on the death certificate. A single five-category race/ethnicity variable was created by treating all those with Hispanic ethnicity as Hispanic, and categorising all non-Hispanics according to race group (black, Asian, American Indian/Alaska Native, white). Age categories employed in standardisation and calculation of age-specific rates and ratios were: less than 1 year, 1 to 4 years, 5 to 14 years, 15 to 24 years, 25 to 34 years, 35 to 44 years, 45 to 54 years, 55 to 64 years, 65 to 74 years, 75 to 84 years and 85+ years. Mortality rates were calculated using bridged-race population estimates from the National Center for Health Statistics [31,32]. Age-adjusted rates were standardised to the population of the United States in 2000. Statistical comparison of medians was accomplished with Wilcoxon-Mann-Whitney tests for independent samples. Differences in rate ratios were compared using chi-squared tests for homogeneity. Unless otherwise noted all reported rates and rate-ratios are age-adjusted. Rate ratios (RR) are the only measure of relative risk reported. Confidence intervals (CI) for rates and rate-ratios were calculated based on variance estimates derived from the Poisson distribution. Time trends were assessed using Poisson regression. Data analysis employed SAS 9.1 (SAS Institute Inc, Cary, NC, USA) and Excel 2003 (Microsoft Corp, Redmond, WA, USA).

## Results

From 1999 to 2005 there were 16,948,482 deaths in the United States. Of these, 1,017,616 were associated with sepsis (6.0% of all deaths). Demographic characteristics, place of death and frequency of comorbidities listed on the death certificates of sepsis decedents are shown in Table 1. Median age for sepsis decedents was 76 years. Males were younger than females: the median age-at-death among men was 74 years compared with 79 years among women (*P* < 0.0001).

**Table 1:** Characteristics of individuals with sepsis-associated deaths in the United States, from 1999 to 2005 (n = 1,017,616)

**Characteristic**	**n (%)**
*Age, years*	
> 1	5794 (0.6)
1 to 4	2341 (0.2)
5 to 14	2421 (0.2)
15 to 24	5410 (0.5)
25 to 34	2314 (1.2)
35 to 44	35,681 (3.5)
45 to 54	76,932 (7.6)
55 to 64	118,272 (11.6)
65 to 74	195,962 (19.3)
75 to 84	311,370 (30.6)
85+	251,076 (24.7)

*Male sex*	474,749 (46.6)

Race/Ethnicity*	
White	749,472 (73.7)
Black	63,731 (17.6)
Hispanic	179,273 (6.3)
Asian	19,228 (1.9)
American Indian/Alaska Native	5912 (0.6)

*Place of death*	
Hospital, clinic or medical centre	883,953 (86.9)
Nursing home	63,900 (6.3)
Residential	57,566 (5.7)
Other or unknown	12,197 (1.2)

*Comorbidities listed on death record*	
Malignant neoplasm	153,531 (15.1)
Diabetes mellitus	117,763 (11.6)
Congestive heart failure	73,198 (7.2)
Chronic renal failure	69,944 (6.9)
Chronic obstructive pulmonary disease	60,765 (6.0)
Hypertension	64,589 (6.3)
Chronic liver disease	28,039 (2.8)
HIV/AIDS	14,599 (1.4)
Chronic alcohol abuse	9739 (1.0)

*Race/ethnicity missing for 43 subjects.

The great majority of sepsis-associated deaths occurred in hospitals, clinics and medical centres (86.9%) and of these 94.6% were inpatients. Other frequent places of death were nursing homes and residences.

During the study period, the average annual crude sepsis-associated mortality rate in the United States was 50.49 deaths per 100,000 persons (95% CI = 50.39 to 50.59). From 1999 to 2005 the crude annual mortality rate increased from 50.14 (95% CI = 49.87 to 50.40) to 52.28 (95% CI = 52.02 to 52.54) deaths per 100,000 persons, corresponding to an annual increase of 0.67% (*P* < 0.0001). After age standardisation the average annual sepsis-associated mortality rate was 50.37 deaths per 100,000 persons (95% CI = 50.28 to 50.47). In contrast to crude mortality, the age-adjusted rate of sepsis-associated mortality decreased by 0.18% per year during the study period (*P* < 0.01).

Race-specific and sex-specific rates of annual sepsis-associated mortality are reported in Table 2. Despite the predominance of women among decedents (53.4%), after controlling for age, men were more likely to experience sepsis-associated death (RR = 1.27, 95% CI = 1.27 to 1.28). The increased risk for men persisted in every age group and among all races. The magnitude of association between male sex and sepsis-associated mortality varied among races (*P* < 0.0001). The association was largest in Asian males, who were 45% more likely than their female counterparts to experience sepsis-associated death (RR = 1.45, 95% CI = 1.41 to 1.49). The effect of male sex on sepsis-related mortality was least apparent in American Indians/Alaska Natives (RR = 1.07, 95% CI = 1.01 to 1.12).

**Table 2:** Average annual race-, sex- and age-specific rates of sepsis-associated mortality in the United States, 1999 to 2005

**Category**	**Females**	**Male**	**Overall**
*Race* Age-adjusted mortality rate per 100,000 (95% CI)*			
White	40.5 (40.3 to 40.6)	51.7 (51.6 to 51.9)	45.1 (45.0 to 45.3)
Black	89.9 (89.3 to 90.5)	117.6 (116.8 to 118.5)	100.9 (100.4 to 101.4)
Hispanic	45.6 (45.1 to 46.2)	58.6 (57.9 to 59.3)	51.3 (50.9 to 51.8)
Asian	29.5 (28.9 to 30.1)	42.8 (41.9 to 43.7)	35.1 (34.6 to 35.6)
American Indian/Alaska Native	54.6 (52.6 to 56.6)	58.2 (55.8 to 60.7)	56.2 (54.7 to 57.7)
*Age *(years) Crude mortality rate per 100,000 (95% CI)			
> 1	18.7 (18.0 to 19.5)	22.7 (22.0 to 23.5)	20.8 (20.24 to 21.3)
1 to 4	1.9 (1.8 to 2.1)	2.3 (2.2 to 2.5)	2.1 (2.0 to 2.2)
5 to 14	0.8 (0.9 to 0.9)	0.9 (0.8 to 0.9)	0.9 (0.8 to 0.9)
15 to 24	1.8 (1.8 to 1.9)	2.0 (1.9 to 2.1)	1.9 (1.9 to 2.0)
25 to 34	4.1 (4.0 to 4.2)	4.7 (4.6 to 4.8)	4.4 (4.3 to 4.5)
35 to 44	10.1 (9.9 to 10.2)	12.8 (12.6 to 13.0)	11.4 (11.3 to 11.5)
45 to 54	23.5 (23.2 to 23.7)	31.9 (31.6 to 32.2)	27.6 (27.4 to 27.8)
55 to 64	55.9 (55.5 to 56.4)	71.1 (70.5 to 71.6)	63.2 (62.9 to 63.6)
65 to 74	133.6 (132.8 to 134.5)	174.2 (173.1 to 175.2)	152.0 (151.4 to 152.7)
75 to 84	309.5 (308.0 to 311.0)	411.4 (409.3 to 413.5)	350.1 (348.9 to 351.4)
85+	745.6 (742.0 to 749.2)	871.5 (865.6 to 877.4)	783.5 (780.4 to 786.5)

*Rates standardised to the US Census 2000 populations.

CI = confidence interval.

We found significant racial disparities in sepsis-associated mortality (*P* < 0.0001). Compared with whites, Asians were less likely to experience sepsis-related death (RR = 0.78, 95% CI = 0.77 to 0.78), while Blacks (RR = 2.24, 95% CI = 2.23 to 2.24), American Indians/Alaska Natives (RR = 1.24, 95% CI = 1.24 to 1.25) and Hispanics (RR = 1.14, 95% CI = 1.13 to 1.14) were more likely than whites to experience sepsis-related death.

Although blacks had the highest rates of sepsis-associated death of all the groups, they also had the sharpest decline in annual age-adjusted sepsis mortality, which fell from 105.97 deaths per 100,000 persons in 1999 (95% CI = 104.64 to 107.30) to 97.00 deaths per 100,000 persons in 2005 (95% CI = 95.81 to 98.20) corresponding to a decline of 1.60% per year (*P* < 0.0001). Sepsis-related mortality rates also fell among Asians (1.34% per year, *P* < 0.01), Hispanics (1.00% per year, *P* < 0.01) and American Indians/Alaska Natives (0.40% per year, *P* = 0.54), although the trend was not significant in American Indians/Alaska Natives. Sepsis-related mortality increased among whites by 0.20% annual during the study period (*P* < 0.01).

**Figure 1:**
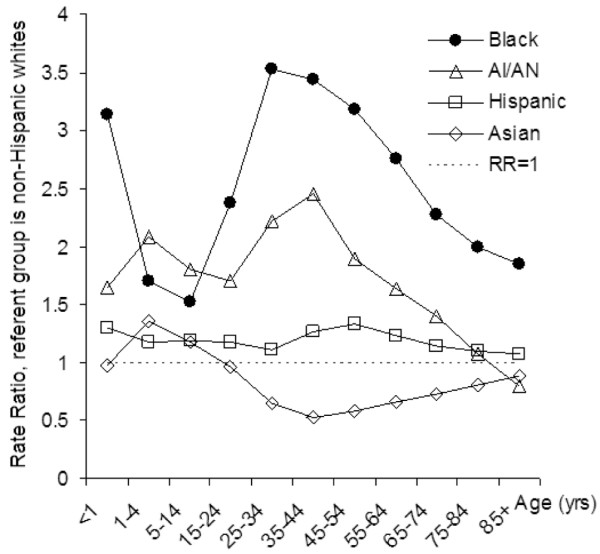
Age-specific rate-ratios for sepsis-associated death by race/ethnicity category in the United States, 1999 to 2005. Non-Hispanic whites were used as the referent group. AI/AN = American Indian/Alaska Native.

Table 2 shows that young children and the elderly experienced the greatest burden of sepsis-related death. The age-specific rate-ratios for sepsis death in racial/ethnic groups are illustrated in Figure 1. Relative to whites, blacks had an increased likelihood for sepsis-associated death at all ages, but their relative risk was greatest in the 35 to 44 years and 45 to 54 years age groups. A similar pattern emerged among American Indians/Alaska Natives. Relative to whites, Asians were more likely to experience sepsis-related death in childhood and adolescence, and less likely during adulthood and older-age. Hispanics were approximately 20% more likely than whites to die of sepsis-related causes across all age groups.

## Discussion

We found that 6% of all deaths in the United States from 1999 to 2005 were sepsis related. Of the sepsis-associated deaths identified in this study, only 22.7% (1.4% of all deaths during the study period) would be attributed to sepsis using the underlying-cause-of-death classification. An underlying-cause-of-death approach to quantifying the burden of sepsis mortality, as used by the National Center for Health Statistics in its annual mortality report, may substantially underestimate the contribution of the syndrome to deaths in the United States [1]. The MCOD approach is particularly appropriate in the analysis of sepsis mortality because the vast majority of sepsis cases occur in elderly individuals and others whose deaths are the result of multiple co-existing ailments [5,10,12-16].

Rates of sepsis-associated mortality were highest among blacks and lowest among Asians. Women were less likely to experience sepsis-related death than men in all race/ethnicity groups. The large majority of sepsis-related deaths occurred in hospitals, clinics and medical centres, but the proportion of death occurring in other settings was significant.

These data confirm previous findings of significant disparities in sepsis mortality between men and women, and among different races [2,18]. Comparable disparities have frequently been reported for incidence of sepsis and severe sepsis [2,4,17-20].

Although several studies have stratified their analysis of sepsis according to race [4,18-20], there is a paucity of data on sepsis-associated mortality rates in Hispanics, American Indians/Alaska Natives and Asians. Earlier studies found that, relative to whites, blacks and other non-whites have a higher incidence of sepsis [4,18-20]. Our data indicates that blacks experience the highest rates of sepsis-related mortality. However, sepsis-related mortality for other non-white groups is heterogeneous. Asians have the lowest rates of all groups including whites. American Indian/Alaska Natives and Hispanics, on the other hand, have intermediate rates of sepsis-related mortality. These differences may reflect distinct rates of incidence and case fatality in these populations and should be studied separately. Our findings may also reflect disparities in access to health care. Encouragingly, rates of sepsis-associated mortality exhibited downward trends in all high-risk groups with the steepest decline for blacks (1.60% per year).

Compared with whites, both American Indians/Alaska Natives and blacks experienced peak relative risk for sepsis-associated death in their thirties and forties. A previous study showed an analogous trend for sepsis incidence among blacks in New Jersey [18]. Another recent study of multi-state hospital inpatient data also noted that the rate of sepsis incidence among blacks diverged from whites at a young age [20]. The comparable rate ratio curves of blacks and American Indians/Alaska Natives indicate a similar early onset of vulnerability for sepsis-related death. The occurrence of parallel patterns of vulnerability in two racially-disparate groups suggests a possible social mechanism for the disparities. However, it is likely that genetic as well as social factors underlie racial disparities in sepsis mortality, and further research will be useful to elucidate such mechanisms. Unfortunately, MCOD data do not provide indicators of socioeconomic status (such as geographical or income data) that would allow an investigation of the relation between such factors and racial disparities in sepsis-associated mortality.

Our study indicates that the age-adjusted rate of sepsis mortality exhibited a very slight downward trend, decreasing by 0.18% per year, from 1999 to 2005. This finding contradicts a study by Dombrovskiy and colleagues which used discharge data from the Nationwide Inpatients Sample to show a rapid increase in the incidence and mortality of severe sepsis between 1993 and 2003 [2]. The investigators found that the national age-adjusted rate of severe sepsis mortality increased annually by 5.6% during this period [2]. Although our study looked at all sepsis-related deaths, while Dombrovskiy and colleagues confined their analysis to severe sepsis, this difference in approaches should not produce conflicting mortality trends. Most decedents included in our study would be expected to have experienced severe sepsis even when organ failure is not listed on the death certificate, because organ failure is the mechanism by which sepsis causes death. Furthermore, reanalysing the data only for those sepsis-associated deaths that had a mention of organ failure on their death record did not alter our findings.

When we restricted our analysis to individuals who were inpatients at the time of their death, and to the period of overlap between our study and that reported by Dombrovskiy and colleagues (1999 to 2003), we found that the rate of sepsis-associated mortality showed a slight annual increase of 0.16% (*P* = 0.01). Although this last result does represent a reversal of trend, our analyses of all sepsis-associated deaths and of sepsis-associated deaths occurring in inpatient facilities demonstrate an essentially flat trend from 1999 to 2003.

Interestingly, the crude rate of severe sepsis mortality reported by Dombrovskiy and colleagues for 2003, the final year of their study, agreed closely with our own estimate for the same year (50.8 and 50.7 deaths per 100,000 population, respectively). However, their estimates for prior years (1999 to 2002) were all substantially lower than mortality rates indicated by our data. The contradictory results may reflect increasing sensitivity in diagnosis and/or coding of severe sepsis in Nationwide Inpatients Sample data or changes in the attribution and coding of sepsis on death certificates. In addition the practice of sampling and weighting may bias estimates based on Nationwide Inpatients Sample data. The disagreement in trends warrants further investigation and underscores the importance of consulting multiple data sources for determining disease burden.

Although our data showed that age-adjusted mortality decreased very slightly over the study period, crude sepsis-related mortality exhibited the opposite trend. The slight decrease in age-adjusted mortality suggests minor improvements in prevention and/or treatment of sepsis. The increasing crude mortality illustrates that population ageing is, and will continue to be, a significant driver of sepsis mortality.

Our data confirms earlier findings that men are at increased risk of sepsis death compared with women, and mirrors previously reported sex disparities in sepsis incidence [2,4,5,17,18]. We also found that the effect of sex on susceptibility to sepsis-related death varied by racial group (*P* < 0.0001). Overall, men were 27% more likely to experience sepsis-associated death. However, the excess risk for Asian men was twice as large, while for American Indians/Alaska Natives being male increased the likelihood of sepsis-related death by only 7%. The reasons underlying these differences cannot be ascertained from MCOD data. Genetic and hormonal factors, as well as varying prevalence of comorbid conditions have been implicated in sex-linked disparities in sepsis, and may all contribute to the pattern of disease observed in our study [17,33,34].

We found that many of the chronic conditions previously associated with sepsis commonly appear on death records which list sepsis as a cause of death. The frequency with which comorbidities are seen on death records that cite sepsis as a cause of death often differs from the reported frequency of these conditions in incident sepsis cases [4,5,17]. This finding is not surprising because data from death records do not reflect all prevalent conditions in decedents [35]. A condition diagnosed in an individual whose death is sepsis-related would not appear on the death record if the clinician completing the certificate were unaware of the diagnosis, or did not believe the condition to be an important contributor to the death. The frequency with which sepsis and a particular comorbidity appear on the same death record does not reflect the frequency of that comorbidity in sepsis decedents; rather it reflects the frequency with which that condition acts as a cause of death when sepsis is also a cause [35].

MCOD data is truly population-based because of the compulsory nature of death reporting in the United States. Our findings indicate that 13.1% of sepsis-associated deaths occur in non-hospital settings. These deaths would not be counted in studies that rely on hospital discharge databases. In addition MCOD data is not vulnerable to sampling bias. However, MCOD data has many limitations characteristic of other administrative datasets.

We cannot directly assess the accuracy of the data. Misclassification of sepsis-related death can result from errors in diagnosis and in the completion of death certificates. Many physicians do not receive formal training in completing death certificates and may disagree on the cause of death [36]. Additionally, errors may occur in coding of death certificate data. As there are no codes for sepsis in ICD-10, we have used codes for septicaemia as a proxy. Septicaemia codes have been used to study sepsis in administrative datasets [2,4,17-19], and Martin and colleagues validated this approach for ICD-9 codes, showing a positive predictive value of 97.7% and negative predictive value of 80.0% [4]. As indicated by the relatively low negative predictive value, the use of septicaemia codes underestimates the burden of sepsis, probably due to the occurrence of sepsis in patients without recognised blood-borne infections. As ICD-10 codes have not been validated for studies of sepsis, our case definition may produce additional misclassification. In addition to error in the numbers of sepsis-related death, rates calculated in our study may also be biased by population estimates. Uncertainty in denominator values results from error in the census count, and from the fact that the population structure for intercensal years is extrapolated [31,32]. An additional limitation in our study is that causative organisms were recorded on death certificates of only 7% of decedents. This omission makes it impossible to reliably assess the relative contribution of various types of microorganisms to sepsis-associated mortality.

## Conclusions

Between 1999 and 2005, sepsis contributed to 6% of all deaths in the United States. Our study shows that the rapid rise in sepsis mortality seen in previous decades has slowed, but that ageing of the United States population continues to drive growth in the overall burden of sepsis-associated mortality. Disparities in sepsis-associated mortality mirror those previously reported for sepsis incidence. However, the age and sex distribution, as well as rates of sepsis-related death, are distinct in Asians, Hispanics and American Indians/Alaska Natives. It is important to study the epidemiology of sepsis in each of these groups because aggregate measures may obscure important differences. The trends in sepsis-associated mortality found in this study contradict reports from previous investigations and must be confirmed.

## Key messages

• Sepsis is a major contributor to mortality in the United States.

• The rapid rise of sepsis mortality seen in previous decades has slowed.

• Population ageing continues to drive growth in the burden of sepsis-associated mortality.

• Sex, age and race/ethnicity disparities in sepsis-associated mortality mirror those previously reported for sepsis incidence.

• The epidemiology of sepsis should be studied individually in racial/ethnic minorities so as to elucidate unique features in each group.

## Abbreviations

CI: confidence interval; ICD: *International Classification of Disease*; MCOD: multiple-cause-of-death; RR: rate ratio.

## Competing interests

The authors declare that they have no competing interests.

## Authors' contributions

AM designed the study, carried out statistical analysis and interpretation of the data, and drafting of the manuscript. FJS conceived of the study, contributed to its design, and aided in the interpretation and drafting of the manuscript.
